# Preclinical Evaluation of [^212^Pb]Pb-ADVC001: A Prostate-Specific Membrane Antigen–Targeted α-Therapy for Prostate Cancer

**DOI:** 10.2967/jnumed.125.269707

**Published:** 2025-11

**Authors:** Feifei Liu, Melissa E. Monterosso, Didier Boucher, Stelle Shakti, Kwong Ching Li, Chanwoo Kim, Amber Prior, Abby Sydes, Amelia T. Soderholm, Nicholas Fletcher, Dewan Akhter, Kristofer Thurecht, William Tieu, Kevin Kuan, Aimee Horsfall, Saawan Kumar, Johannes Koehbach, Edward Hammond, Anna Karmann, Stephen Rose, Gary Li, Simon Puttick, Thomas Kryza

**Affiliations:** 1 AdvanCell Pty Ltd., Sydney, New South Wales, Australia; and; 2 Centre for Advanced Imaging and Australian Institute for Bioengineering and Nanotechnology, The University of Queensland, St Lucia, Queensland, Australia

**Keywords:** targeted α-therapy, PSMA, ^212^Pb, theranostics, prostate cancer, preclinical

## Abstract

Prostate-specific membrane antigen (PSMA)–directed radiopharmaceutical therapies continue to improve treatment outcomes in patients with metastatic castration-resistant prostate cancer. Here, we report the in vitro and in vivo characterization of a PSMA-targeted therapy (ADVC001) specifically designed for targeted α-therapy with ^212^Pb. **Methods:** The binding affinity to PSMA was determined by PSMA enzymatic assays and by radioligand binding assays using PSMA-high prostate cancer (PC) cells. In vitro cytotoxicity against PC cell lines with high and medium PSMA expression was evaluated using clonogenic, metabolic, and imaging-based cytotoxic assays. Pharmacokinetics and biodistribution were assessed using PSMA-high subcutaneous tumor xenografts. In vivo single-dose and multidose efficacy was assessed in subcutaneous PC xenograft models expressing various levels of PSMA. **Results:** A high binding affinity to PSMA was observed for ADVC001 with nanomolar inhibitory concentration of 50% values. In cellular assays, [^212^Pb]Pb-ADVC001 (^212^Pb-ADVC001 hereafter for simplicity) exhibited specific cytotoxic activity against PSMA-expressing cells with nanomolar effective concentration of 50% values. In vivo biodistribution of ^212^Pb-ADVC001 in the PC3-PIP xenograft model revealed rapid and persistent tumor uptake, fast renal clearance, and low retention in normal tissues. Single-dose efficacy studies of ^212^Pb-ADVC001 (0.46 MBq) showed improved survival compared with [^177^Lu]Lu-PSMA-I&T (^177^Lu-PSMA-I&T hereafter for simplicity) (20 MBq) treatment. In a multidose experiment, 2 doses of ^212^Pb-ADVC001 (0.5 MBq) significantly increased median survival (86 d vs. 45.5 d, *P* < 0.05) compared with 2 doses of ^177^Lu-PSMA-I&T (15 MBq). Treatment with ^212^Pb-ADVC001 (0.5 MBq) after initial ^177^Lu-PSMA-I&T (15 MBq) relapse showed an enhanced survival benefit (59.5 d). In a C4-2 xenograft model with medium-level PSMA expression, single doses of 0.3, 0.8, and 1.1 MBq of ^212^Pb-ADVC001 significantly extended median survival to 34, 57, and 62.5 d, compared with untreated cohorts (16 d). All treatments were well tolerated. **Conclusion:** The preclinical results support the clinical development of ^212^Pb-ADVC001 as a targeted α-therapy for the treatment of patients with PC.

## Visual Abstract

**Figure:**
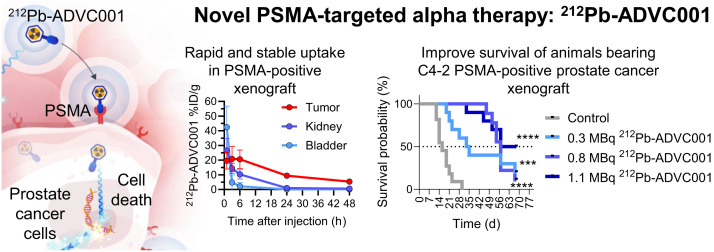

Prostate-specific membrane antigen (PSMA), a type II membrane protein, is highly expressed in most prostate tumors (1). The PSMA protein is a membrane-bound glutamate carboxy peptidase II protein encoded by the folate hydrolase gene. It has a short N-terminal cytoplasmic tail, a single membrane-spanning helix, and an extracellular domain. The extracellular region comprises 95% of the protein and is a suitable cell-surface target for drug development (2). On binding of a ligand to the protein, internalization occurs, allowing for endocytosis of bound drugs such as PSMA-targeted radiopharmaceuticals, resulting in increasing concentration of radioactivity within PSMA-expressing tumor cells (3).

PSMA has an attractive expression profile with high expression in most prostate cancers and metastases and minimal expression in normal tissues. A low level of expression is seen in normal prostate tissue, kidneys, salivary and lacrimal glands, duodenum, brain, and intestines (4). PSMA-targeted radiopharmaceuticals have been investigated with diagnostic imaging isotopes such as ^99m^Tc, ^68^Ga, and ^18^F and with β- (^177^Lu and ^161^Tb) or α-emitting therapeutic isotopes (^225^Ac, ^211^At, and, more recently, ^212^Pb). The Food and Drug Administration approval and subsequent commercialization of the first PSMA-targeted radiopharmaceutical therapy, [^177^Lu]Lu-PSMA-617, has changed the practice of treatment of metastatic castration-resistant prostate cancer (mCRPC) with a demonstrated survival benefit in patients who received taxane-based chemotherapy and a strong overall safety profile (5).

AdvanCell is developing the investigational medicinal product [^212^Pb]Pb-ADVC001 (^212^Pb-ADVC001 hereafter for simplicity), a PSMA-targeting ligand radiolabeled with ^212^Pb intended for the treatment of patients with prostate cancer. ADVC001 is based on the well-established radioligand PSMA-I&T or DOTAGA-(I-y)fk(Sub-KuE) (6). PSMA-I&T has been used extensively in the management of patients with mCRPC with both ^225^Ac and ^177^Lu radionuclides and has shown clinical tolerability and efficacy (6–8). ^212^Pb is an in vivo generator of the high-energy α-particle–emitting radionuclide ^212^Bi. Targeted α-therapy using ^212^Pb delivers high-energy α-radiation directly to cancer cells, causing irreparable DNA damage (9). ^212^Pb-ADVC001 is currently being evaluated in a phase 1b/2a clinical trial (NCT05720130) for the treatment of patients with mCRPC (10). Here, we report preclinical studies investigating the affinity, cytotoxicity, biodistribution, and efficacy of ^212^Pb-ADVC001 undertaken to support its translation to first-in-human studies.

## MATERIALS AND METHODS

### Radiochemistry

[^212^Pb]PbCl_2_ in 0.05 M hydrochloric acid (manufactured by AdvanCell) was freshly prepared on the day of the experiment and added to a solution of ADVC001 buffered with sodium acetate (pH 5.5). The reaction solution was agitated at 25 °C for 20 min, and then a sample was spotted onto an instant thin-layer chromatography strip (glass microfiber chromatography paper impregnated with silicic acid; Agilent Technologies) and developed in an aqueous solution of EDTA (25 mM, pH 5–6). ^212^Pb activity on the top and bottom halves of the strip were individually measured using the 238-keV peak on a NaI detector connected to a multichannel analyzer to determine radioisotope incorporation. The resulting ^212^Pb-ADVC001 was formulated with sodium ascorbate and 0.1 mg/mL of DTPA, and the radiochemical purity was assessed by reverse-phase high-performance liquid chromatography using a LabLogic FlowRAM equipped with a 1-mm photomultiplier tube NaI detector.

### Animal Studies

All animal studies were conducted under the approval of The University of Queensland Molecular Bioscience Animal Ethics Committee and the Australian Code for the Care and Use of Animals for Science Purposes (2023/AE000708, 2023/AE000645). BALB/c nude mice were chosen for experiments involving ^177^Lu-PSMA-I&T to avoid artificial toxicity associated with the hypersensitivity of NOD scid gamma (NSG) animals. After cell injection or treatment, animals were monitored at least 3 times a week with general health checks and measurement of tumor volume and body weight to determine tumor growth and animal survival.

### Biodistribution

Human prostate cancer PSMA-high PC3-PIP cells (1 × 10^6^ cells) were resuspended in 100 µL of a 1:1 mixture of phosphate-buffered saline (PBS) and Matrigel (Corning) and were injected into the right flank of NSG mice under isoflurane anesthesia (male, 8–10 wk old, NSG: NOD.Cg-*Prkdc*^scid^ *ll2rg*^tm1Wjl^/SzJ/Ozarc). Once tumors reached an average volume of approximately 200 mm^3^, animals received 0.1 mL of treatment solution (1 MBq/0.5 µg of ^212^Pb-ADVC001) diluted in formulation solution (50 mg/mL sodium ascorbate and 0.1 mg/mL DTPA administered through the tail vein using a 0.5-mL 29-G insulin syringe. Injection time and the effective dose injected (dose loaded in syringe minus dose left in syringe after injection) for each mouse were recorded. At predetermined time points, mice were sacrificed by CO_2_ inhalation and dissected. The organs and tissues were rinsed in PBS (except for blood, serum, bone marrow, carcass) and placed in preweighed γ-counter tubes. Specific ^212^Pb activity was measured on a PerkinElmer Wizard 2480 γ-counter (Revvity). Raw counts per minute were corrected for background from each rack, transformed into activity using the counter efficiency, and decay-corrected to injection time. Decay-corrected activity was normalized to the effective dose administered to each animal (percent of injected dose [%ID]) and to organ weight (percent of injected dose per gram [%ID/g]).

### Single-Dose Efficacy Using PC3-PIP Cells

PC3-PIP cells (1 × 10^6^ cells) were resuspended in 100 µL of a 1:1 mixture of PBS and Matrigel and were injected into the right flank of male BALB/c-*Foxn1^nu^*/Ozarc (BALB/c) nude mice under isoflurane anesthesia (8–10 wk old). Once tumors reached an average volume of approximately 200 mm^3^, animals received 0.1 mL of treatment solution administered through the tail vein using a 0.5-mL 29-G insulin syringe. Treatment solutions consisted of 1 µg of nonradioactive ADVC001 (*n* = 5), 1 µg/20 MBq of ^177^Lu-PSMA-I&T (*n* = 5), 1 µg/115 kBq of ^212^Pb-ADVC001 (*n* = 5), and 1 µg/463 kBq of ^212^Pb-ADVC001 (*n* = 5) diluted in formulation solution. After treatment, animals were monitored at least 3 times a week with general health checks and measurement of tumor volume and body weight to determine tumor growth and animal survival. The human equivalent dose (HED) was calculated using the following formula: HED = injected dose in animal × (animal weight/human weight)^0.33^ (11). Values were calculated assuming a 20–25-g mouse and a 70–75-kg human.

### Multidose Efficacy Using PC3-PIP Cells

PC3-PIP cells (1 × 10^6^ cells) were resuspended in 100 µL of a 1:1 mixture of PBS and Matrigel and were injected into the right flank of male BALB/c nude mice under isoflurane anesthesia (8–10 wk old). Once tumors reached an average volume of approximately 400 mm^3^, animals received a first treatment consisting of 0.1 mL of treatment solution administered through the tail vein using a 0.5-mL 29-G insulin syringe: 1 µg of nonradioactive ADVC001 (*n* = 8), 15 MBq (1 µg) of ^177^Lu-PSMA-I&T (*n* = 12), and 1 µg/500 kBq of ^212^Pb-ADVC001 (*n* = 7) diluted in formulation solution. On tumor recurrence (∼14 d after first treatment), animals received a second treatment dose as above. Doses used in this experiment were chosen to represent approximately 50% of the clinical HED.

### Single-Dose Efficacy Using C4-2 Cells

Human prostate cancer PSMA-positive C4-2 cells (4 × 10^6^ cells) were resuspended in 100 µL of a 1:1 mixture of PBS and Matrigel and were injected in the right flank of NSG mice under isoflurane anesthesia (male, 8–10 wk old). Once tumors reached an average volume of approximately 200 mm^3^, animals received 0.1 mL of treatment solution administered through the tail vein using a 0.5-mL 29-G insulin syringe. Treatment solutions consisted of 0.5 µg of nonradioactive ADVC001 (*n* = 7), 300 kBq of ^212^Pb-ADVC001 (0.5 µg/MBq, *n* = 10), 800 kBq of ^212^Pb-ADVC001 (0.5 µg/MBq, *n* = 9), and 1,100 kBq of ^212^Pb-ADVC001 (0.5 µg/MBq, *n* = 10) diluted in formulation solution.

### Statistical Analyses

Data were analyzed using GraphPad Prism. Results are presented as mean ± SD except when otherwise indicated. The Mann–Whitney test was used for comparing 2 groups from in vivo assays. For mouse treatment assays, the Kruskal–Wallis test was used for comparisons involving more than 2 groups, whereas survival was compared using Mantel–Cox and Gehan–Breslow–Wilcoxon tests.

## RESULTS

### Binding Affinity of ADVC001 and ^212^Pb-ADVC001

**FIGURE 1.**
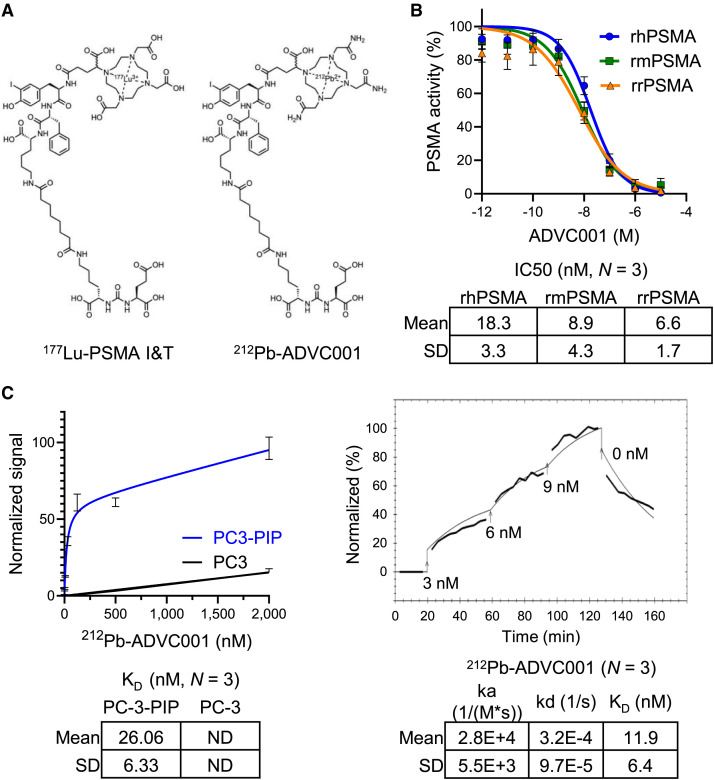
Evaluation of binding affinity of ADVC001 and ^212^Pb-ADVC001. (A) Chemical structures of ^177^Lu-PSMA-I&T and ^212^Pb-ADVC001. (B) Inhibitory activity of ADVC001 against recombinant human PSMA (rhPSMA), recombinant mouse PSMA (rmPSMA), and recombinant rat PSMA (rrPSMA) enzymes. Data are presented as average ± SD from 3 independent experiments. (C) Left: steady-state binding of ^212^Pb-ADVC001 onto PSMA-high PC3-PIP cells determined using γ-counter. Data are presented as average ± SD from 3 independent experiments. Right: kinetic binding of ^212^Pb-ADVC001 onto PSMA-high PC3-PIP cells determined using ligand tracer. Data are presented as average ± SD from 3 independent experiments. IC_50_ = half-maximal inhibitory concentration; ka = association rate constant; K_D_ = equilibrium dissociation constant; kd = dissociation rate constant; ND = no data.

The radiopharmaceutical used in this study, ADVC001, is based on the well-established clinical-stage radioligand PSMA-I&T (Fig. 1A). For PSMA-I&T PET imaging (^68^Ga) and therapy (^177^Lu or ^225^Ac), DOTA-based chelators are conjugated to PSMA-I&T via an appropriate linker to complex these metal ions. For ADVC001 therapy (^212^Pb), a DO3AM-based chelator is used. When conjugated with ^212^Pb, the chemical structures of ^212^Pb-ADVC001 and ^177^Lu-PSMA I&T are similar, with the difference between the 2 drugs being the bivalent metal chelator used to attach the radioisotope (Fig. 1A).

Results of the binding affinity of ADVC001 using recombinant PSMA proteins and PSMA-expressing cells are given in Figure 1. The inhibitory activity of the ADVC001 precursor against the enzymatic activity of recombinant human, mouse, and rat PSMA was determined in a competition assay with a fluorogenic substrate (Fig. 1B) (12). Increasing concentrations of ADVC001 inhibit human, mouse, and rat PSMA with high potency, with average half-maximal inhibitory concentration values of 18.3 ± 3.3, 8.9 ± 4.3, and 6.6 ± 1.7 nM, respectively (mean ± SD, *n* = 3). To confirm the specific binding of ADVC001 to PSMA in a cellular system, we assessed the accumulation of ^212^Pb-ADVC001 in prostate cancer cells with or without PSMA expression in steady-state and kinetic experiments (Fig. 1C). After background correction and normalization for maximum uptake, a strength of interaction of 26.06 ± 6.33 nM was calculated for ^212^Pb-ADVC001 against PSMA-high PC3-PIP cells (mean ± SD, *n* = 3; Fig. 1C). The strength of interaction for PSMA-negative PC3 cells was not calculated (Fig. 1C). In kinetic experiments, the dynamic binding of ^212^Pb-ADVC001 to PSMA-high PC3-PIP cells was measured using a ligand tracer (13). ^212^Pb-ADVC001 showed an average association rate constant of 2.8 × 10^4^ ± 5.5 × 10^3^ (1/(M·s)) (mean ± SD, *n* = 3). The strength of interaction of ^212^Pb-ADVC001 for PSMA receptors was found to be 11.9 ± 6.4 nM (mean ± SD, *n* = 3), further demonstrating a strong in vitro ligand–receptor interaction (Fig. 1C).

### In Vitro Cytotoxicity

**FIGURE 2.**
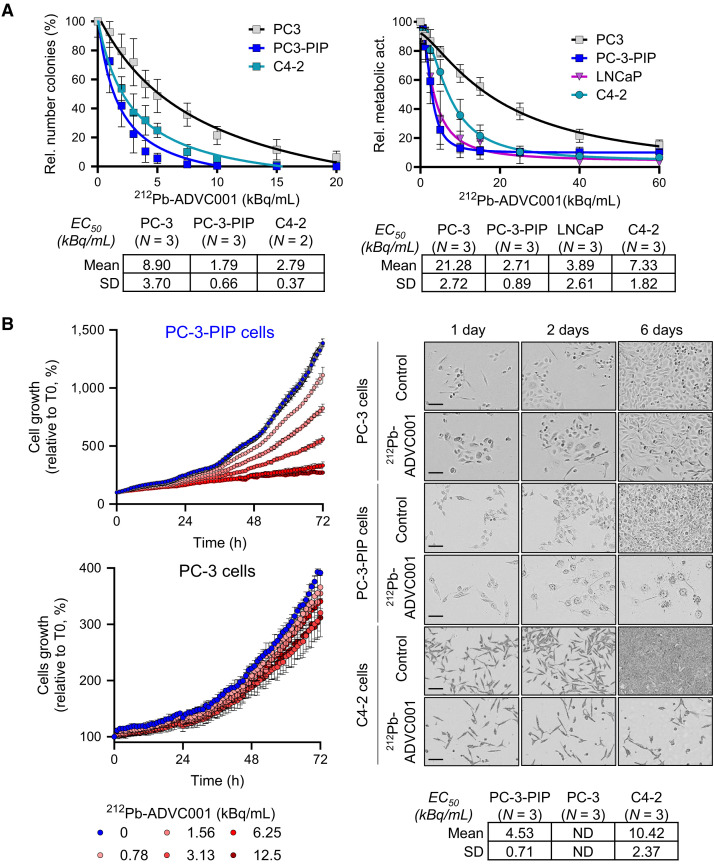
Evaluation of cytotoxic activity of ^212^Pb-ADVC001 against prostate cancer cells. (A) Left: clonogenic assay performed with PC3, PC3-PIP, and C4-2 cells. Cells were seeded at ultralow density after treatment with ^212^Pb-ADVC001 (0–20 kBq/mL). Colonies formed were counted after 10–14 d of culture. Data are presented as average ± SD from *n* = 2–3 independent experiments. Right: metabolic assay performed on PC3, PC3-PIP, LNCaP, and C4-2 cells 5 d after treatment with ^212^Pb-ADVC001 (0–60 kBq/mL). EC_50_ are presented as average ± SD from *n* = 3 independent experiments. (B) Left: time-lapse imaging of PC3 and PC3-PIP cells treated with ^212^Pb-ADVC001 (0–12.5 kBq/mL). Data are expressed as relative cell number compared with number of cells at treatment start. Right: representative images of cells 5 d after treatment with 5 and 15 kBq/mL of ^212^Pb-ADVC001 for PC3 and PC3-PIP cells and C4-2 cells, respectively (scale bar = 100 µm); ^212^Pb-ADVC001 EC_50_ determined by time-lapse imaging (average ± SD, *n* = 3 independent experiments). act. = activity; Rel. = relative; T0 = beginning of treatment.

To confirm the PSMA-specific cytotoxic activity of ^212^Pb-ADVC001, we measured the impact of ^212^Pb-ADVC001 activities (0–60 kBq/mL) on the colony formation capacity, metabolic activity, and cellular growth of prostate cancer cells expressing various levels of PSMA (Supplemental Figs. 1A and 1B; supplemental materials are available at http://jnm.snmjournals.org). The clonogenic assay showed that ^212^Pb-ADVC001 inhibited colony formation by 50% (half-maximal effective concentration [EC_50_]) at an average dose of 1.79 ± 0.66 kBq/mL (*n* = 3) and 2.79 ± 0.37 kBq/mL (*n* = 2) for PC3-PIP (PSMA^hi^, engineered to express PSMA) and C4-2 (PSMA^int^, endogenous PSMA expression) cells, respectively (Fig. 2A, left; Supplemental Fig. 1C). PC3 parental cells, which do not express PSMA, were significantly less sensitive to ^212^Pb-ADVC001 with an EC_50_ of 8.9 ± 3.7 kBq/mL (*n* = 3; Fig. 2A, left). In the metabolic assay, ^212^Pb-ADVC001 displayed a potent cytotoxic activity with an average EC_50_ of 2.71 ± 0.89 kBq/mL (*n* = 3), 3.89 ± 2.61 kBq/mL (*n* = 3), 7.33 ± 1.82 kBq/mL (*n* = 3), and 21.28 ± 2.72 kBq/mL (*n* = 3) for PC3-PIP, LNCaP, C4-2, and PC3 cells, respectively (Fig. 2A, right). The image-based cytotoxic assay also showed that ^212^Pb-ADVC001 displayed potent cytotoxic activity against PSMA-positive prostate cancer cells with an average EC_50_ of 4.53 ± 0.71 kBq/mL (*n* = 3) and 10.42 ± 2.37 kBq/mL (*n* = 3) for PC3-PIP and C4-2 cells, respectively, whereas the EC_50_ was not calculated for PC3 because of the low level of cell death (Fig. 2B; Supplemental Fig. 2). Notably, under the tested conditions, PC3 cells exhibited slower growth than PC3-PIP cells, which may influence the calculated EC_50_ values. As shown in Supplemental Figure 1D, the EC_50_ values were also dependent on treatment duration. Nevertheless, under both treatment conditions (4 and 24 h), the differential response between cancer cells expressing varying levels of PSMA was maintained.

### Biodistribution and Pharmacokinetic Evaluation

**FIGURE 3.**
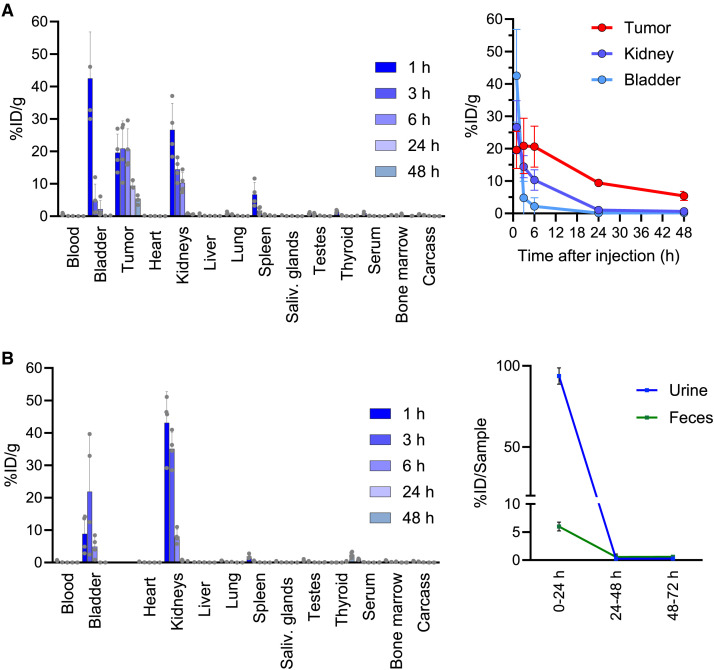
Biodistribution and pharmacokinetic of ^212^Pb-ADVC001. Biodistribution analysis of ^212^Pb-ADVC001 in NSG mice bearing subcutaneous PSMA-high PC3-PIP tumor (A) and in non–tumor-bearing mice (B). (A) Animals received 1 MBq of ^212^Pb-ADVC001 (0.5 µg/MBq) intravenously and were sacrificed at specific time points (1, 3, 6, 24, and 48 h). Organs were harvested and weighted, and their activity was measured on γ-counter. Data are expressed as average ± SD %ID/g from *n* = 4 per time point and organ. Left: %ID/g per organ per time point. Right: kinetic of activity measured in bladder, kidney, and tumor. (B) Arc:Arc(s) male mice received 0.5 MBq of ^212^Pb-ADVC001 (0.5 µg/MBq) intravenously and were processed as in A. Left: %ID/g per organ per time point. Right: Pharmacokinetics analysis (*n = 3* cages of 2 animals per cycle, average ± SD %ID). Saliv. = salivary.

The biodistribution of ^212^Pb-ADVC001 (1 MBq, 0.5 µg/MBq) was evaluated in male NSG mice bearing PSMA-high PC3-PIP tumors and in non–tumor-bearing Arc:Arc(s) male mice at 1, 3, 6, 24, and 48 h after injection (*n* = 4/time point). As shown in Figure 3A, ^212^Pb-ADVC001 reached maximum uptake in tumor tissue within 3 h after injection (20.85 ± 4.27 %ID/g) with excellent tumor retention out to 48 h after injection (5.38 ± 0.71 %ID/g). ^212^Pb-ADVC001 showed fast clearance from the blood (<1 %ID/g by 1 h after injection) and bladder (<1 %ID/g by 24 h after injection) (Fig. 3A; Supplemental Fig. 3A; Supplemental Table 1). Kidneys were the normal organ with the highest uptake (26.63 ± 4.10 %ID/g at 1 h after injection) followed by rapid clearance with approximately 1 %ID/g remaining 24 h after injection. Tumor/kidney ratios were 0.74, 1.44, 2.22, 9.24, and 8.66 at 1, 3, 6, 24, and 48 h, respectively (Supplemental Table 2). Blood levels were low (0.63 ± 0.14 %ID/g) at 1 h and almost undetectable by 3 h (<0.1 %ID/g). The fast clearance of ^212^Pb-ADVC001 was further supported by the biodistribution and the urine and feces analysis from non–tumor-bearing mice treated with ^212^Pb-ADVC001 (Fig. 3B; Supplemental Fig. 3B; Supplemental Table 3). These studies confirmed the fast clearance from the blood of ^212^Pb-ADVC001 and showed that 99.75% of the injected dose was found in the excreta within the first 24 h after dose (93.77 ± 5.00 %ID in urine and 5.98 ± 0.78 %ID in feces) (Fig. 3B).

Dosimetry assessments from biodistribution data in PSMA-high tumor-bearing mice were performed to estimate the absorbed radiation dose in human organs for starting dose selection of ^212^Pb-ADVC001 in first-in-human trials. The dosimetry methodology and equations are provided in the supplemental materials and methods (14–17). Based on the maximum allowable dose of 23 Gy for kidneys (14,18), this threshold would be reached at 2.47 GBq/66.7 mCi for males, assuming a relative biological effectiveness (RBE) of 5. The estimated absorbed doses at clinically relevant and maximum administered activities of ^212^Pb-ADVC001 and dose limits for each organ are listed in Table 1.

**TABLE 1.** Dosimetry Analysis of ^212^Pb-ADVC001*

	Absorbed dose (Gy) at clinically relevant activity				Maximum administered activity	
Organ/tissue	60.00 MBq (1.62 mCi)	120.00 MBq (3.24 mCi)	180.00 MBq (4.86 mCi)	240.00 MBq (6.49 mCi)	2,467.52 MBq (66.69 mCi)	Dose limit (Gy)
Kidneys	0.559	1.119	1.678	2.237	23.00	23
Pancreas	0.012	0.024	0.036	0.048	0.492	N/A
Liver	0.013	0.026	0.039	0.052	0.533	30
Stomach^†^	0.024	0.049	0.073	0.097	0.999	45
Lungs^‡^	0.020	0.041	0.061	0.081	0.837	20
Urinary bladder wall	0.614	1.229	1.843	2.457	25.266	65
Heart wall	0.007	0.015	0.022	0.029	0.301	26
Red marrow	0.012	0.023	0.035	0.046	0.477	2
Salivary glands	0.011	0.022	0.033	0.044	0.451	20
Tumor^§^	1.614	3.228	4.842	6.456	66.376	NA

* Dosimetry analysis performed on the basis of biodistribution presented for human doses ranging from 60 to 240 MBq.

† Stem cell layer.

‡ Alveolar–interstitial.

§ 10.24-cm diameter.

NA = not available.

### Antitumor Efficacy

**FIGURE 4.**
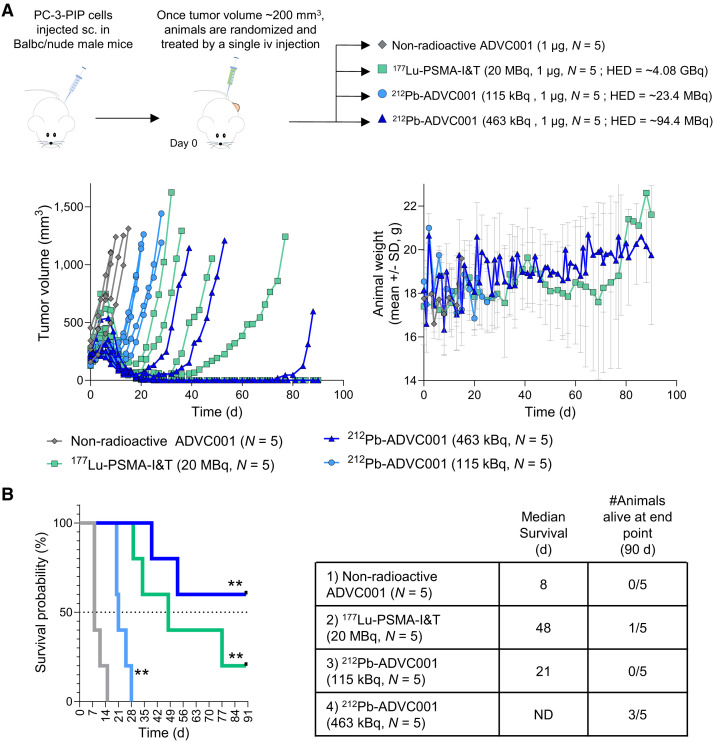
Evaluation of ^212^Pb-ADVC001 single doses efficacy in PSMA-high PC model. ^212^Pb-ADVC001 was evaluated in nude mice bearing subcutaneous (sc.) PSMA-high PC3-PIP tumor. Animals were treated with single intravenous (iv) dose of nonradioactive ADVC001 (1 µg), ^212^Pb-ADVC001 (115 or 463 kBq, 1 µg), or 20 MBq of ^177^Lu-PSMA-I&T (1 µg) (*n* = 5 per group). (A) Tumor growth monitoring (individual animal) and animal body weight (mean ± SD, *n* = 5). (B) Survival curves and summary of median survival and number of animals alive at end of study. Mantel–Cox test and Gehan–Breslow–Wilcoxon test with Bonferroni multiple comparison correction for 3 comparisons versus ADVC001 (**P < 0.005).

First, we compared the antitumor efficacy of ^212^Pb-ADVC001 with that of ^177^Lu-PSMA-I&T in the PSMA-high PC3-PIP xenograft model in BALB/c nude mice (Fig. 4). Tumor monitoring showed that ^212^Pb-ADVC001 reduced tumor progression in a dose-dependent manner without significantly affecting body weight (Fig. 4A). ^212^Pb-ADVC001 improved median survival from 8 d for those treated with nonradioactive ADVC001 to 21 d for those that received 115 kBq of ^212^Pb-ADVC001 and to greater than 90 d for those treated with 463 kBq of ^212^Pb-ADVC001 (3/5 animals alive at 90 d, including 2 without tumor). For comparison, animals treated with ^177^Lu-PSMA-I&T (20 MBq) had a median survival of 48 d (Fig. 4B). A toxicity study performed in naïve BALB/c nude mice using ^212^Pb-ADVC001 (600 kBq, 1 µg) or ^177^Lu-PSMA-I&T (20 MBq, 1 µg) showed no significant acute toxicity as measured by animal body weight, organ weight, and blood hematologic parameters for up to 42 d (Supplemental Fig. 4).

**FIGURE 5.**
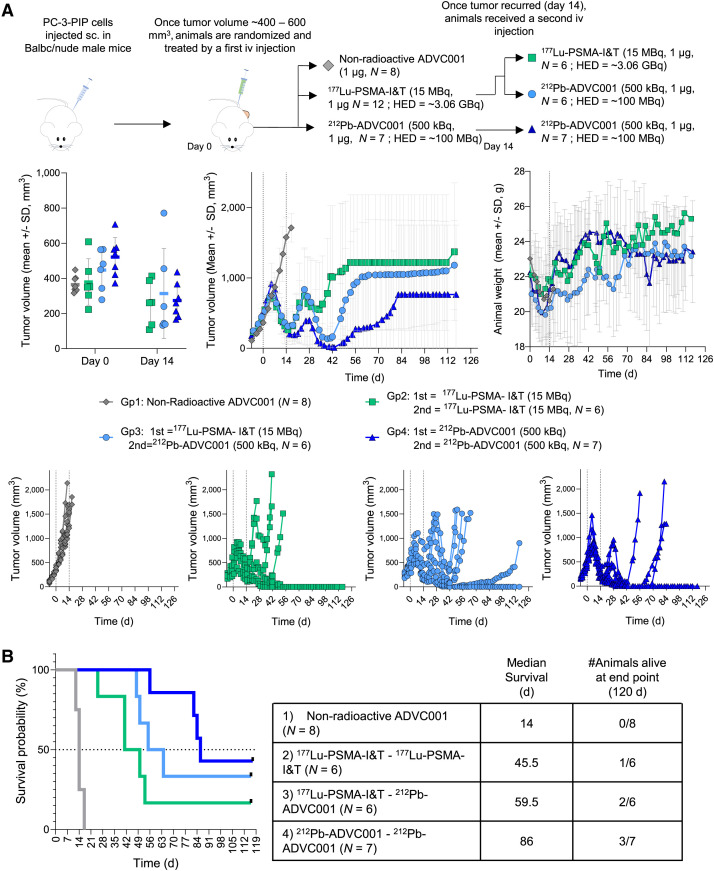
Evaluation of in vivo efficacy of ^212^Pb-ADVC001 on ^177^Lu-PSMA therapy recurring tumor. ^212^Pb-ADVC001 was evaluated in nude mice bearing subcutaneous (sc.) PSMA-high PC3-PIP tumor. Animals were first treated with intravenous (iv) dose of 15 MBq of ^177^Lu-PSMA-I&T (1 µg) (*n* = 12 per group) or 500 kBq of ^212^Pb-ADVC001 (1 µg), and tumor growth was monitored. Once tumor recurred, animals received second dose of either 15 MBq of ^177^Lu-PSMA-I&T (1 µg) (*n* = 6 per group) or 500 kBq of ^212^Pb-ADVC001 (1 µg, *n* = 6). (A) Left: tumor volume at day 0 (first treatment) and day 14 (second treatment). Middle: tumor growth monitoring (mean ± SD and individual animal). Right: animal body weight monitoring (mean ± SD). (B) Survival curves and summary of median survival and number of animals alive at end of study.

We then evaluated the efficacy of ^212^Pb-ADVC001 on PSMA-high prostate cancer tumors recurring after ^177^Lu-PSMA therapy. To generate a ^177^Lu-PSMA recurrent tumor model, BALB/c nude mice bearing large subcutaneous PSMA-high PC3-PIP xenografts (average volume of ∼400 mm^3^) received an intravenous dose of ^177^Lu-PSMA-I&T (15 MBq, 1 µg, HED ∼3.1 GBq) (Fig. 5). This lower dose of ^177^Lu-PSMA-I&T, representing about 50% of a human-equivalent clinical dose, induced tumor regression for up to 14 d, after which tumors recurred. Animals bearing ^177^Lu-PSMA-I&T recurrent tumors then received a second treatment with either ^177^Lu-PSMA-I&T (15 MBq, 1 µg) or ^212^Pb-ADVC001 (500 kBq, 1 µg, HED ∼100 MBq), each representing about 50% of a human-equivalent clinical dose (Fig. 5A, left). For comparison, a separate group received 2 doses of ^212^Pb-ADVC001 (500 kBq, 1 µg), with the first dose also leading to tumor recurrence in most animals 14 d after treatment, similar to ^177^Lu-PSMA-I&T (15 MBq, 1 µg).

Tumor growth monitoring showed that ^212^Pb-ADVC001 (500 kBq, 1 µg) produced a similar antitumor effect in both naïve and ^177^Lu-PSMA-I&T recurrent tumors. In contrast, ^177^Lu-PSMA-I&T exhibited reduced efficacy in ^177^Lu-PSMA-I&T recurrent tumors compared with naïve ones (Fig. 5A, middle). No effect on animal body weight was observed during this experiment (Fig. 5A, right). Animals that received ^212^Pb-ADVC001 (500 kBq, 1 µg) as a second dose showed an improved median survival of 59.5 d compared with 45.5 d for those that received a second dose of ^177^Lu-PSMA-I&T. Two doses of ^212^Pb-ADVC001 (500 kBq, 1 µg) further extended median survival to 86 d, with 3 of 7 animals alive at the end of the study (120 d; Fig. 5B).

**FIGURE 6.**
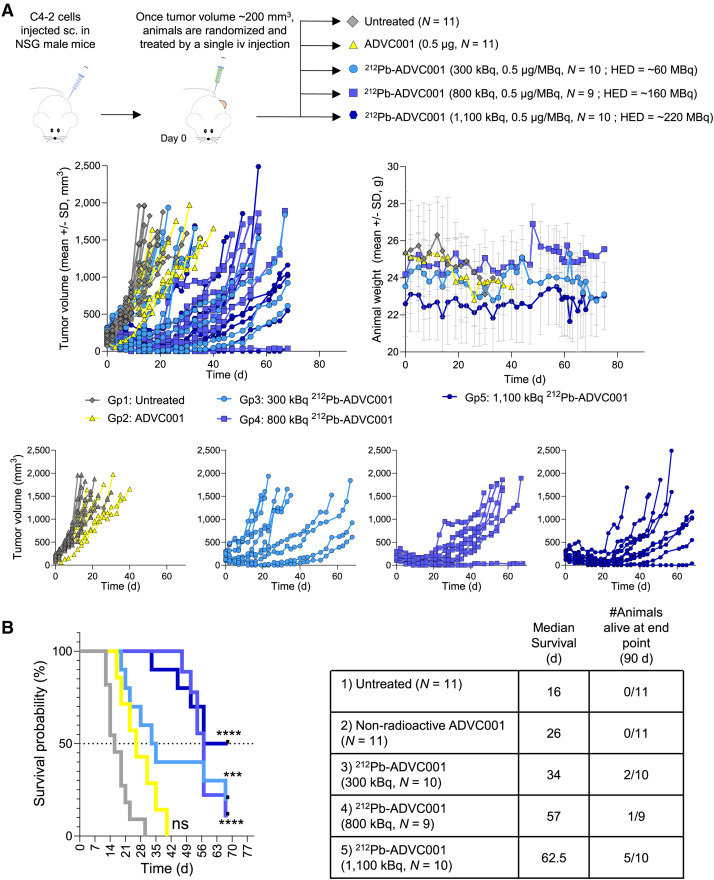
Evaluation of in vivo efficacy of single dose of ^212^Pb-ADVC001 in PSMA-intermediate prostate cancer model. ^212^Pb-ADVC001 was evaluated in NSG mice bearing subcutaneous (sc.) PSMA-positive C4-2 tumor. Animals were treated with single intravenous (iv) dose of nonradioactive ADVC001 (0.5 µg) or ^212^Pb-ADVC001 (300, 800, or 1,100 kBq, 0.5 µg/MBq). (A) Tumor growth monitoring (individual animal) and animal body weight (mean ± SD, *n* = 9–11). (B) Survival curves and summary of median survival and number of animals alive at end of study. Mantel–Cox test and Gehan–Breslow–Wilcoxon test with Bonferroni multiple comparison correction for 3 comparisons versus untreated. ***P ≤ 0.001 and ****P ≤ 0.0001. Gp = group; ns = not significant.

Finally, to confirm whether ^212^Pb-ADVC001 would demonstrate a strong antitumor efficacy in prostate cancer lesions expressing an intermediate level of PSMA, a single-dose efficacy study was performed in the PSMA-positive C4-2 xenograft model in NSG mice (Fig. 6) that express a lower level of PSMA than PC3-PIP cells (Supplemental Fig. 1). ^212^Pb-ADVC001 was found to reduce tumor growth in a dose-dependent manner without significantly affecting body weight (Fig. 6A). The low dose of ^212^Pb-ADVC001 (300 kBq, 0.5 µg/MBq) reduced tumor growth in about 50% of the animals, whereas both 800 and 1,100 kBq of ^212^Pb-ADVC001 (0.5 µg/MBq) further suppressed tumor growth out to 21 d in all treated animals (Fig. 6A). ^212^Pb-ADVC001 improved median survival from 26 d for those treated with nonradioactive ADVC001 to 34, 57, and 62.5 d, respectively, for those animals that received 300, 800, or 1,100 kBq of ^212^Pb-ADVC001 (Fig. 6B).

## DISCUSSION

Therapeutic radiopharmaceuticals have the potential to provide a paradigm shift in the treatment of cancer. ^177^Lu-PSMA-617 is the first Food and Drug Administration–approved radiopharmaceutical therapy for patients with mCRPC (5,19). ^177^Lu-PSMA-617 plus best standard of care demonstrated a median survival benefit in the post–taxane-based chemotherapy setting of 4 mo (5). Unfortunately, ^177^Lu-PSMA–targeting agents are ineffective in approximately 30% of patients, with most patients ultimately progressing because of radioresistance (20). In contrast, ^212^Pb-PSMA–based radioligands present an alternative approach and benefit from the use of a high-energy α-particle–emitting payload which causes irreparable double-strand DNA damage to cancer cells (21). Other advantages include a 10.6 h half-life which may reduce potential off-site toxicity and the advantage of being able to produce clinical doses of ^212^Pb via generator production (22). ^212^Pb-ADVC001 is a ^212^Pb-PSMA–targeted α-therapy currently being evaluated in a phase 1/2 clinical trial (10). The results of the preclinical work undertaken here in terms of target binding, cytotoxicity, biodistribution, and efficacy of ^212^Pb-ADVC001 provide overwhelming support for the translation to first-in-human studies.

We demonstrated that ^212^Pb-ADVC001 specifically binds to PSMA in vitro, with binding affinities in the nanomolar range, consistent with previously reported half-maximal inhibitory concentration values for ^177^Lu-PSMA-I&T (23). On binding and internalization, ^212^Pb-ADVC001 showed potent cytotoxic effects on PSMA-expressing cells. The cytotoxicity level was found to be consistent with the receptor expression level in vitro. Biodistribution studies showed rapid tumor accumulation and high tumor retention of ^212^Pb-ADVC001 in vivo. The radiopharmaceutical cleared quickly through the kidney and urinary tract, with minimal retention in normal tissue. These findings are consistent with the pharmacokinetics of previously reported ^212^Pb-labeled low-molecular-weight compounds for targeted radiopharmaceutical therapy of prostate cancer (24).

^212^Pb-ADVC001 shows impressive anticancer efficacy against PSMA-positive prostate cancer models. A single-dose in vivo efficacy study showed significant tumor regression and prolonged survival in human PSMA-positive prostate cancer xenograft models after treatment with ^212^Pb-ADVC001, with some animals exhibiting a long-term survival benefit. In the multidose efficacy study, we showed that ^212^Pb-ADVC001 has comparable effectiveness in both naïve (group 4) and ^177^Lu-treated (group 3) prostate cancer tumors, indicating that ^212^Pb-PSMA therapy may be a potential treatment option for patients who progress after ^177^Lu-PSMA therapy, though clinical validation is still required. As not all patients present high PSMA expression, we investigated the efficacy of ^212^Pb-ADVC001 in a cell model endogenously expressing PSMA (C4-2 xenograft model). Again, ^212^Pb-ADVC001 exhibited a favorable efficacy profile in an expected dose-dependent manner. Taken together, the efficacy studies demonstrated significant suppression of tumor growth at low injected doses compared with the maximum tolerated dose levels determined by our dosimetry study.

^212^Pb-ADVC001 displayed a rapid clearance from the blood and low uptake in normal organs. There was no obvious toxicity observed based on assessment of body weight, organ weight, and hematology parameters. The human estimated absorbed doses for ^212^Pb-ADVC001 were extrapolated using the biodistribution studies in mice. α-particles have a high linear energy transfer compared with photons and β-particles, leading to different biologic effects (25). Therefore, the absorbed doses need to be adjusted to reflect their biologic impact. The RBE is often used to account for differences in biologic damage caused by different types of ionizing radiation. RBE values between 3 and 7 have been suggested for α-particle dosimetry (15), although an agreed value of RBE for α-particles remains uncertain. For this study, an RBE of 5 was used, as recommended by the Food and Drug Administration (22). This dosimetry study identified the kidneys as the dose-limiting organ for adult males. Given the current 23-Gy external beam radiation therapy–defined threshold, extrapolation of the animal dosimetry data supports the administration of up to 9 doses of 240 MBq in patients. These findings present a favorable dosimetry profile for ^212^Pb-ADVC001 within the clinical trial setting. Combined, the high tolerable dose range and excellent biodistribution profile in terms of rapid tumor accumulation with rapid clearance from normal organs indicate a favorable therapeutic clinical window for ^212^Pb-ADVC001.

## CONCLUSION

We have developed and evaluated a PSMA-targeted α-radiopharmaceutical for the treatment of prostate cancer. In vivo studies have shown that this radiopharmaceutical exhibited rapid uptake and retention in PSMA-positive tumors and fast clearance from blood and normal organs. A single administration of ^212^Pb-ADVC001 demonstrated significant tumor regression and extended survival in human prostate cancer xenograft models in mice. This radiopharmaceutical holds great promise and offers a potentially wide therapeutic window for the treatment of patients with prostate cancer.

## DISCLOSURE

All studies herein were financially supported by AdvanCell. Feifei Liu, Melissa Monterosso, Didier Boucher, Stelle Shakti, Kwong Ching Li, Chanwoo Kim, Amber Prior, Abby Sydes, Amelia Soderholm, William Tieu, Kevin Kuan, Aimee Horsfall, Saawan Kumar, Johannes Koehbach, Edward Hammond, Anna Karmann, Stephen Rose, Simon Puttick, and Thomas Kryza are employees of AdvanCell. William Tieu, Kevin Kuan, Aimee Horsfall, Anna Karmann, Stephen Rose, Gary Li, Simon Puttick, and Thomas Kryza hold shares or options in AdvanCell. William Tieu, Kevin Kuan, and Simon Puttick are inventors on a patent application directed to ADVC001. Dewan Akhter, Nicholas Fletcher, and Kristofer Thurecht are researchers from the Center for Advanced Imaging (CAI) and Australian Institute for Bioengineering and Nanotechnology (AIBN), The University of Queensland. The Translational Research Institute is financially supported by the Australian Federal Government. ^212^Pb used in this research was generated by the AdvanCell process using ^228^Th supplied by the U.S. Department of Energy Isotope Program, managed by the Office of Science for Isotope R&D and Production. Dosimetry analyses were performed by RAPID (Radiopharmaceutical Imaging and Dosimetry, LLC, USA). Kristofer Thurecht acknowledges funding from the Australian Research Council for the ARC Research Hub for Advanced Manufacture of Targeted Radiopharmaceuticals and the National Imaging Facility, a national facility established under the National Collaborative Research Infrastructures Scheme. No other potential conflict of interest relevant to this article was reported.

## Notes

KEY POINTS **QUESTION:** Does the use of ^212^Pb-ADVC001 have potential for the treatment of patients with PSMA-positive mCRPC? **PERTINENT FINDINGS:** ^212^Pb-ADVC001 showed high affinity and PSMA-specific cytotoxicity in multiple in vitro systems, with high tumor uptake and fast renal clearance with minimal accumulation in normal organs in vivo. These properties subsequently contributed to significant and durable anticancer efficacy without observable toxicity in animal models of PSMA-positive prostate cancer. **IMPLICATIONS FOR PATIENT CARE:** This innovative radiopharmaceutical therapy exhibits significant potential and a broad therapeutic window for treating prostate cancer.
